# Healthcare personnel frequently have positive severe acute respiratory syndrome coronavirus 2 (SARS-CoV-2) antigen tests 5 days or more after diagnosis of coronavirus disease 2019 (COVID-19)

**DOI:** 10.1017/ice.2022.21

**Published:** 2022-02-08

**Authors:** Usha Stiefel, Davinder Bhullar, Trina F. Zabarsky, Natalie F. Palmieri, Kimberly D. Diaz, Maria M. Torres-Teran, Curtis J. Donskey

**Affiliations:** 1 Infectious Diseases Section, Louis Stokes Cleveland VA Medical Center, Cleveland, Ohio; 2 Case Western Reserve University School of Medicine, Cleveland, Ohio; 3 Personnel Health Department, Louis Stokes Cleveland VA Medical Center, Cleveland, Ohio; 4 Infection Control Department, Louis Stokes Cleveland VA Medical Center, Cleveland, Ohio; 5 Department of Nursing, Louis Stokes Cleveland VA Medical Center, Cleveland, Ohio; 6 Research Service, Louis Stokes Cleveland VA Medical Center, Cleveland, Ohio

*To the Editor—*During the coronavirus disease 2019 (COVID-19) pandemic, healthcare facilities have had to balance the goals of preventing healthcare-associated transmission of severe acute respiratory syndrome coronavirus 2 (SARS-CoV-2) and maintaining adequate staffing.^1^ The emergence of the highly transmissible omicron variant has greatly exacerbated staffing shortages due to frequent infections in unvaccinated and vaccinated personnel.^2^ In response, the Centers for Disease Control and Prevention (CDC) recently provided modified guidance to mitigate healthcare staffing shortages.^2,3^ Under contingency strategies, personnel with mild-to-moderate or asymptomatic COVID-19 infection may return to work 5 days after symptom onset if afebrile and improving, either with or without a test to confirm resolution of the infection.^3^

The rationale for allowing healthcare personnel to return to work after 5 days is that the highest risk for transmission is the period 2 days before and 3 days after symptom onset.^3–5^ However, the duration of shedding of viable virus particles is unclear for the omicron variant, and the frequency of positive antigen tests 5 or more days after onset of illness is not known. Such information is urgently needed because positive antigen tests have been shown to correlate relatively well with shedding of viable virus and transmission risk.^6–9^ Here, we examined the percentage of healthcare personnel with positive antigen tests 5 or more days after diagnosis of COVID-19.

The evaluation was conducted as a quality assurance activity by staff from the Infectious Diseases Section and Personnel Health Department at the Louis Stokes Cleveland VA Medical Center. Beginning January 3, 2022, the facility began performing SARS-CoV-2 antigen testing of personnel with asymptomatic or mild-to-moderate but improving COVID-19 at 5 or more days after diagnosis as a contingency measure to mitigate staffing shortages.^2^ The day of diagnosis was day 0. Personnel were asked to report for testing on day 5 or on their next scheduled workday between days 6 and 9; after day 10, personnel could return to work with no testing. Anterior nares swabs were collected under supervision of laboratory personnel. The BinaxNOW COVID-19 Ag Card (Abbott) was used to detect viral nucleocapsid protein directly from the nasal swab samples according to the manufacturer’s instructions. The number of days since the positive diagnostic test and the COVID-19 vaccination status of the personnel were recorded. The percentage of healthcare personnel with positive antigen test results was graphed, stratified by the number of days since diagnosis of COVID-19. We used the Fisher exact test to compare the percentages of positive antigen tests at days 5–10 after diagnosis for unvaccinated versus fully vaccinated and/or boosted employees. For a subset of 71 employees, personnel health records were reviewed to determine whether respiratory symptoms were present at the time of diagnosis.

**Fig. 1.**
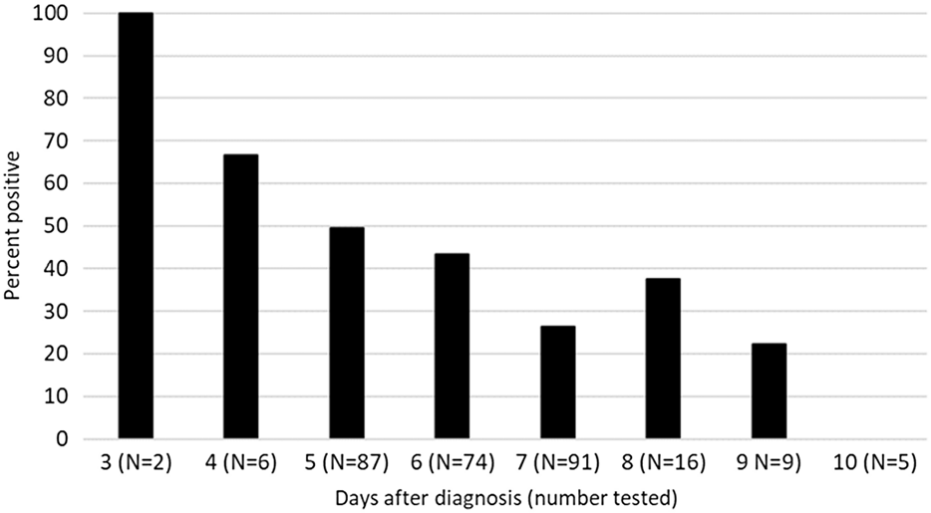
Percentage of healthcare personnel with positive antigen test results by number of days since diagnosis.

Of 290 total employees tested between days 3 and 10 after COVID-19 diagnosis, 113 (39%) had positive antigen tests. The percentage of employees with positive antigen tests decreased as the number of days after diagnosis increased (Fig. 1). At day 5 after diagnosis, 43 (49%) of 87 antigen tests were positive. For tests collected between days 5 and 10 after diagnosis, there was no difference in the percentage of positive tests for unvaccinated versus fully vaccinated and/or boosted employees: 19 (38.8%) of 49 versus 87 (38.3%) of 227 (*P* = 1.0). For the 71 employees whose records were reviewed, 65 (91.5%) had respiratory symptoms at the time COVID-19 was diagnosed and 6 (8.5%) were asymptomatic. Also, 19 (29.2%) of 65 symptomatic employees and 0 of 6 (0%) asymptomatic employees had positive antigen test results, respectively. There were no suspected transmissions of SARS-CoV-2 to coworkers from employees returning to work after a negative antigen test.

Under CDC-recommended contingency strategies, healthcare personnel with asymptomatic or mild-to-moderate COVID-19 may return to work after at least 5 days have passed since symptom onset with or without testing to confirm resolution of infection.^3^ However, many experts recommend that all individuals with COVID-19 have a negative test if isolation is to be discontinued before a full 10 days after a positive test.^9^ Our findings provide support for that recommendation because positive antigen tests were common among healthcare personnel tested 5–9 days after diagnosis. If such testing is not completed, the CDC recommends stringent adherence to measures, such as facemasks and social distancing, to minimize the risk of transmission to patients or coworkers.^1,2^

Our study had several limitations. The assessment was conducted in a single hospital using 1 type of antigen test. Additional data are needed for other antigen test kits. Sequencing was not performed to determine the SARS-CoV-2 variant infecting the study personnel. However, the assessment occurred in the context of widespread (>90%) omicron variant transmission in our region. Assessment of symptoms was completed for only a subset of employees. Further studies are needed to determine whether asymptomatic individuals are less likely to have positive antigen results 5 or more days after diagnosis than symptomatic individuals. The day of diagnosis was considered day 0 for our assessment, whereas the CDC has recommended that day 0 should be the day that symptoms first appeared.^3^ Because many personnel may have been tested 1 or more days after symptom onset, our results may underestimate the duration of positive antigen tests for facilities that conduct testing based on the timing of symptom onset.

Finally, further studies are needed to determine whether persistent antigen positivity on day 5 or later after diagnosis is associated with culture of viable virus and risk for transmission.
